# Through the rear view mirror: a content evaluation of the journal of Chiropractic & Osteopathy for the years 2005–2008

**DOI:** 10.1186/1746-1340-16-14

**Published:** 2008-11-13

**Authors:** Ian D Coulter, Raheleh Khorsan

**Affiliations:** 1 RAND/Samueli Chair for Integrative Medicine, RAND Corp., Santa Monica, USA; 2 Senior Health Policy Researcher, RAND Corp., Santa Monica, USA; 3 Professor, School of Dentistry, University of California Los Angeles, Los Angeles, USA; 4 Research Consultant, Office of Supported and Institutional Research, Southern California University of Health Sciences, Whittier, USA; 5 Research Associate, Military Medical Research, Samueli Institute, Corona del Mar, USA; 6 Research Associate, Integrative Medicine, Samueli Institute, Corona del Mar, USA

## Introduction

The first edition of what was to become the journal, *Chiropractic & Osteopathy *was first published in 1992.*Chiropractic & Osteopathy*, the official journal of the Chiropractic & Osteopathic College of Australasia (COCA), was known then as the COMSIG Review (Chiropractors and Osteopaths Musculo-Skeletal Interest Group). This changed to the Australasian Chiropractic and Osteopathy journal in 1996 and in 2005 became Chiropractic & Osteopathy.

The rationale for the journal was stated clearly in 2005: [1] "There is an imperative for both professions to research the principles and claims that underpin them, and *Chiropractic & Osteopathy *provides a scientific forum for the publication of such research." The intent of the journal is stated as "*Chiropractic & Osteopathy *will encompass all aspects of evidenced-based information that is relevant to chiropractors, osteopaths and related health care professionals. [1] The Journal accepts for publication: primary research, case reports, reviews (both systematic and narrative), commentaries, database articles, debate articles, hypotheses, methodology articles, short reports and study protocols.

It is therefore an appropriate time to look back over the last three years and assess the extent to which the journal has achieved these goals. Ultimately you are what you do not what you say, however, in journals it might be more correct to say you are what you are allowed to be. That is, the content of a journal is driven by what is submitted, by feedback from peer-reviewers, what the readership will read and purchase, and by what the editors would like to see the journal publish. In this article we will examine the data presented by what has been published to draw some conclusions about the likely impact of the journal and perhaps the future. While the latter are speculative, they are based on the data of the journal itself. So we might claim it is grounded speculation.

From the above we can infer some objectives the journal hoped to accomplish. The journal was to be:

1. a journal for both chiropractic and osteopathy

2. an international journal

3. a journal that publishes evidence regarding the claims of chiropractic and osteopathy

4. a journal that publishes evidence collected across a wide range of sources from primary research to reviews to case studies

5. a journal that that encourages commentaries on important issues

## Methods

We reviewed all abstracts for articles published from 2005 to 2008.

Using an *a priori *classification scheme, two reviewers categorized each article published from the inception of the journal in April 2005 to September 12, 2008. We categorized the domain of knowledge, methods, topical content, year of publication, country of origin, whether articles represented core values of chiropractic or osteopathic health care. These were then categorized in numerous ways to generate data tables used in this paper. We then reviewed articles as exemplars of the content for categories. We reconciled differences by discussion and consensus.

## Results

Eighty-three (83) full length articles were published in the journal from April 2005 to September 12, 2008. The results are organized in terms of the five objectives of the journal as originally stated and we examine the evidence to see if the objective has been achieved or not.

### 1. A journal for both chiropractic and osteopathy

The first important distinction pertains to the name of the journal. Although it is called *Chiropractic & Osteopathy *the predominance of articles are focused on chiropractic. Of the 83 articles published since 2005, only 4 were either on osteopathy or written by an osteopath as the lead author. (See Table 1) Overwhelmingly the articles and the authors have been related to chiropractic. To the extent that the objective was for both chiropractic and osteopathy to publish, the journal is not really meeting that objective.

**Table 1:** Type of therapy

Complementary and Alternative medicine (CAM) = 1
Chiropractic = 34
Chiropractic plus another therapy = 6
*Not Specific/Other = 32
Spinal Manipulative Therapy (SMT) = 9
Osteopathy = 1

*Not specific to one particular type of therapy (i.e. spans multiply health interventions).

### 2. An International journal

Originally the journal was to be an Australasian journal. This was later changed to an international journal with an international editorial board. In terms of the country of origin the papers published fall into the following categories: USA 40, Australia 22, Canada 9, and Europe 9 (including UK). Of the 83 articles about 3 articles involved the collaboration of the USA, Australia, Canada and Europe. (See Table 2) While on the one hand this distribution reflects a desire to be international, the dominance from the United States in a journal that originates in Australasia is problematic. The lack of any paper from New Zealand, Oceania including islands of the Pacific Ocean, and Asia would also seem to imply that the journal is not meeting at least one of the original objectives. The results probably reflect that the United States in terms of numbers of chiropractors, chiropractic colleges, chiropractic patients, chiropractic research still does dominate internationally. However, given that this dominance has waned considerably over the last decade or more, it is still surprising to see that papers originating from the United States equal all other papers combined.

**Table 2:** Country of article origin

Australia = 22
Canada = 9
Europe(including UK) = 9
USA = 40
Unknown = 3

### 3. Publishes evidence regarding the claims of chiropractic and osteopathy

If we assume that above all else this objective must at least include evidence for clinical practice/therapy we can test it by looking at the clinical studies published.

From April 2005 to September 2008, *Chiropractic & Osteopathy *has published a total of 83 articles of which 31 are primary research studies (including basic sciences, program evaluations, clinical trials, surveys, and other designs). Of the 9 clinical research studies, the conditions/problems focused mainly on are: 1) low back pain, 2) chronic back pain, 3) neck pain (including chronic neck pain), 4) thoracic pain, 5) cervical pillar hyperplasia and 6) degenerative joint disease, and 7) idiopathic scoliosis. (See Table 3)

**Table 3:** Type of study design

***83 Total Articles***
Case Report = 12
Case Series = 6
Commentary/Expert Opinion = 12
Methodology = 1
Editorial = 1
Primary Research = 31 of which
2 = basic science
2 = program evaluation
2 = cohort study
6 = other study
9 = clinical trial
10 = survey
Narrative Review = 16
Systematic Review = 4

The 18 case studies (including single case reports and case series) on clinical conditions for their part, focused on cancer, post-traumatic upper cervical subluxation, bilateral synovial chondromatosis of the ankle, aberrant shoulder movement, gout in the wrist, xiphodynia, upper extremity radicular and referred pain, right hamate hook fracture, low back pain, malignant spine pain, hearing, scoliosis, shoulder, cervical stenosis, hamstring injury.

Two conclusions can be made about these results. The first is that when primary clinical studies are submitted to the journal the focus is very much neuromusculoskeletal. Case studies on the other hand evidence a much broader range of problems. In one sense the case studies seem to be more likely used to highlight the more unusual cases that can be encountered in a chiropractic/osteopathic clinic rather than the "run-of-mill" cases. To this extent they red flag things a chiropractor or osteopath might encounter in practice. It might also reflect the instructions for authors that actually emphasis on the unusual rather than typical cases.

Again if we assume that the most powerful evidence is from the most rigorous studies then another way of looking at objective 3 is to analyze the study designs used in the original research articles. The designs included: surveys 10, trials 9, cohort studies 2, descriptive studies 2, basic science studies 2, program evaluation 2, methods research 1, and focus groups 1. With the exception of the trials, these design types cannot establish rigorous evidence of efficacy. Surveys establish at best correlative evidence for effectiveness. Cohort studies and descriptive studies tend to be suggestive studies in terms of establishing outcomes. Basic science projects address biological mechanisms and are more important in providing explanatory information than efficacy but are unlikely to be published in this journal. Only the trials could provide definitive evidence for efficacy. The percent of clinical trials published over three years was 12% of the studies published. In comparison *Journal of Manipulative and Physiological Therapeutics *(JMPT) over a three year period had about 15% of its publications in the category of clinical trials. [2] On this standard therefore the journal is performing really well.

### 4. Publishes evidence collected across a wide range of sources from primary research to reviews to case studies

Examining the published articles in terms of the categories the journal will accept for publication, the breakdown is as follows: primary research (31), reviews (both systematic and narrative) (20), case reports (18), commentaries (12). In this review, we were unable to find a definite distinction between most commentaries and debate articles. For the other categories: hypotheses, short reports and study protocols no articles have been published. So if the aim was to publish "evidence" the emphasis on primary studies would be expected. To this extent therefore the journal is meeting its fourth objective.

### 5. Encourages commentaries on important issues

Since commentaries might also be a major way that journals can express issues they feel are of concern to their readers or which fall within the objectives of the journal we also looked at them. In the period we reviewed there were a total of 12 commentaries. Only one pertained to osteopathy alone and dealt with cranial osteopathy. Two were critiques or reinterpretation of previously published literature reviews, one on SMT and another on kinesiology. We found a commentary on the role and emergence of the biopsychosocial model in modern medical literature and health care settings with respect to the management of hypothyroidism and a commentary on the establishment of the Chiropractic & Osteopathic College of Australasia in Queensland. The other 7 focused on chiropractic. Four of the latter focused on broader aspects of the chiropractic profession; professionalism; chiropractic education and wellness standards; chiropractic sports medicine in Australia; and a "chiropracticness" test. It would seem therefore that the chiropractors are much more likely to express themselves in commentaries in the journal than are osteopaths.

## Readership

*Chiropractic & Osteopathy *is an open access, peer-reviewed online journal. It is indexed in PubMed, PubMed Central, Scopus, the Manual Alternative and Natural Therapy Index System (MANTIS), Index to Chiropractic Literature (ICL), and Google Scholar. Internationally the journal is also in repositories at the University of Potsdam in Germany, at INIST in France and in e-Depot, the National Library of the Netherlands' digital archive of all electronic publications. Currently, the journal is also participating in the British Library's e-journals pilot project, and plans to deposit copies of all articles with the British Library.

Currently *Chiropractic & Osteopathy *is part of the BioMed Central, an independent open access publisher. The Chiropractic & Osteopathic College of Australasia has agreed to cover the cost of article-processing charges for all manuscripts submitted before March 2009. This will enable *Chiropractic & Osteopathy *to remain an international open access journal without charge to authors during this time. Articles published with BioMed Central are immediately and permanently available online. Unrestricted use, distribution and reproduction in any medium is permitted, provided the article is properly cited.

Another way of looking at the impact of the journal is to review what articles readers are accessing online. Looking at the 10 most accessed single articles of all time in the journal they are as follows: case report (24,388 hits); case report (19,607); review (19,440); review (18,264); debate (16,236); case report (15,468); debate (13,679); review (13,098); research (12,400); case report (12,109). Clearly case reports are by far the most accessed. These case reports are on scoliosis, shoulder, cervical stenosis, hamstring injuries respectively. The other important results are that in the top ten most accessed articles only one is original research. Three out of the ten most accessed articles are on: 1) a review article on non-surgical decompression injury; 2) a debate article/commentary on theoretical definition of subluxation; and 3) a commentary on chiropractic as spine care. Other most accessed article include reviews, one on reliability and validity of muscle testing and one on the biopsychosocial model and hypothyroidism.

## Discussion

Looked at in terms of their objectives, the journal has had some successes and some mixed successes. Clearly the journal provides a much stronger focus on chiropractic than it does on osteopathy. In fact the small number of articles specifically about osteopathy would seem to imply that a rethinking of the title of the journal may be in order. Although much of the content of this journal may be relevant to osteopathic practice, anyone accessing this journal with the expectation of finding many articles by osteopaths or specifically about osteopathy is likely to be disappointed. The focus of the articles is overwhelmingly chiropractic.

Similarly, with the objective to be an international journal. We do not have data to determine what percentage of those who publish in Australasia on chiropractic publish in this journal. Perhaps this number of articles from Australian authors is a significant achievement. What we can say is that a journal that is dominated by articles whose origin is the US has some challenges if it wants to be an international journal for chiropractic. The lack of any papers we could find as having an Australasia source is also a challenge for the journal.

The objective to publish evidence about osteopathy and chiropractic has not been achieved for the former but has had some success for chiropractic. Around 37% of the articles published fall into the category primary research studies. There were 20 reviews (both systematic and narrative) which can also be interpreted as providing evidence of efficacy and/or effectiveness. So the verdict here might be mixed success.

The one area where the journal has met its objective very well is to publish a wide range of research. It has published trials, cohort studies, case studies, reviews, basic science reports, surveys, descriptive studies, program evaluations.

The number of commentaries (12) is not a huge number. They are almost entirely focused on chiropractic issues. They are also on a very diverse set of topics which reflects that the journal is being used to express commentaries over a wide range of concerns. This can be viewed as a positive feature of the journal.

The data from the hits on line are interesting in that they reflect that those accessing the journal seem to have a different objective in mind than perhaps the editors do. Overwhelmingly they are accessing either a case report or a review or a debate. None of the original research articles has as many hits as these. It would seem the readership is accessing clinical cases more than anything else. At the very least their priorities are different. The journal published 31 articles on primary research and only 18 case reports.

## Conclusion

The results therefore pose some challenges to the editorial staff. If the journal is to continue to title itself Chiropractic & Osteopathy it must find some new method of attracting osteopaths to publish in the journal on osteopathic subjects. The other option might be to change the title.

Similarly if it is to be a truly international journal it should reflect this more in its contents. A journal that is dominated by articles originating from the US might not be very successful in portraying itself as truly international or as the Australasian journal for chiropractic. If this is the objective again some means have to be found to address this issue.

Last but not least, given what the readers are accessing online some thought might be given to expanding the number of case studies that are published. The journal is not the only journal publishing original research articles in chiropractic so publishing original research articles does not distinguish it too well in the market place.
